# Usutu Virus: An Arbovirus on the Rise

**DOI:** 10.3390/v11070640

**Published:** 2019-07-12

**Authors:** Ferdinand Roesch, Alvaro Fajardo, Gonzalo Moratorio, Marco Vignuzzi

**Affiliations:** 1 Institut Pasteur, Viral Populations and Pathogenesis Unit, CNRS UMR 3569, 75015 Paris, France; 2 Laboratorio de Virología Molecular, Centro de Investigaciones Nucleares, Facultad de Ciencias, Universidad de la República, Montevideo 11400, Uruguay; 3 Laboratorio de Inmunovirología, Institut Pasteur de Montevideo, Montevideo 11400, Uruguay

**Keywords:** USUV, Usutu, WNV, flavivirus, emergence

## Abstract

The Usutu virus (USUV) is a flavivirus that is drawing increasing attention because of its potential for emergence. First isolated in Africa, it was introduced into Europe where it caused significant outbreaks in birds, such as in Austria in 2001. Since then, its geographical distribution has rapidly expanded, with increased circulation, especially in the last few years. Similar to West Nile virus (WNV), the USUV enzootic transmission cycle involves *Culex* mosquitoes as vectors, and birds as amplifying reservoir hosts, with humans and other mammals likely being dead-end hosts. A similarity in the ecology of these two viruses, which co-circulate in several European countries, highlights USUV’s potential to become an important human pathogen. While USUV has had a severe impact on the blackbird population, the number of human cases remains low, with most infections being asymptomatic. However, some rare cases of neurological disease have been described, both in healthy and immuno-compromised patients. Here, we will discuss the transmission dynamics and the current state of USUV circulation in Europe.

## 1. Introduction

The dramatic outbreaks and global spread of the chikungunya virus (CHIKV) and the Zika virus (ZIKV) illustrate the danger that emerging arboviruses represent. Given the plethora of mosquito-transmitted viruses, which have been identified to date, and the known ability of RNA viruses to mutate rapidly to adapt to new environments, it is likely that other arboviruses will emerge in the next decades. The Usutu virus (USUV), an arbovirus from the *Flaviviridae* family, genus *Flavivirus*, has recently garnered a great deal of attention from the scientific community, particularly since its emergence in Europe. Similar to other flaviviruses, its (+)-strand RNA genome of 11,064 nucleotides encodes a single polyprotein of 3,434 amino acids that is subsequently cleaved into structural (C, prM and E) and non-structural (NS1, NS2A, NS2B, NS3, NS4A, NS4B and NS5) proteins. The natural life cycle of USUV involves *Culex* mosquitoes as its main vector, and multiple bird species as a natural viral reservoir. To date, USUV has been mostly associated with disease in birds, with spectacular episodes of mass mortality occurring in Central Europe; but it can also infect humans, and in some rare cases, it can be associated with neurological complications.

In this review, we will summarize the current knowledge on USUV, with particular emphasis on the increased circulation in Europe in recent years, the dynamics of transmission, and the human clinical cases described to date. 

## 2. Introduction of USUV in Europe and Genetic Diversity

USUV belongs to the Japanese encephalitis virus (JEV) antigenic complex, together with West Nile virus (WNV) and Murray Valley encephalitis virus (MVEV). It was isolated for the first time in 1959 from a *Culex neavei* mosquito caught near the Usutu river in Swaziland [1]. Since then, the virus has continuously circulated within Africa, having been detected in Senegal, Uganda, Central African Republic, Nigeria, Burkina Faso, and Côte d’Ivoire (reviewed in [2]). USUV was observed in Europe for the first time in Vienna in 2001 [3], where it was responsible for massive outbreaks in blackbirds (*Turdus merula)* and gray owls *(Strix nebulosa)*. A retrospective study analyzing dead birds conducted in the Tuscany region of Italy showed that USUV has been circulating in Europe since 1996 [4].

**Figure 1:**
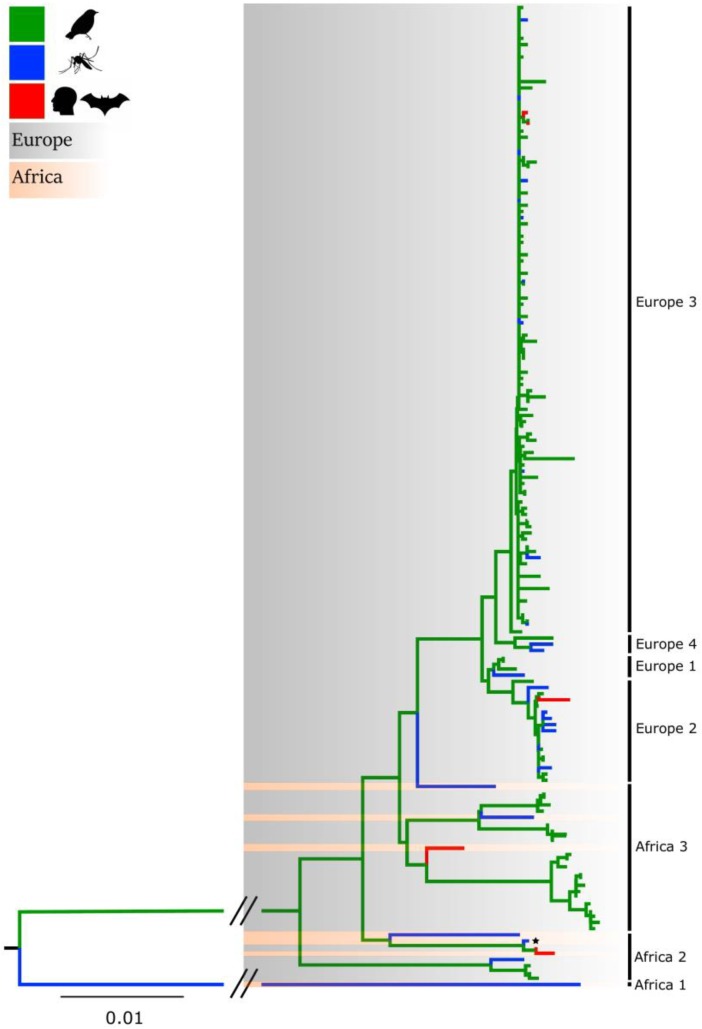
Maximum-likelihood phylogenetic tree analysis of the NS5 gene of USUV strains (*n* = 158) circulating worldwide. Strains isolated from birds, mosquitoes and mammals are indicated in green, blue and red, respectively. African and European variants are shaded in pink and gray, respectively. The prototype SAAR-1776 strain is indicated by a star. The bar at the bottom of the tree denotes evolutionary distance, as number of base substitutions per site. The interrupted branches (indicated by oblique lines) were shortened by 50% for better graphic representation. The list of USUV strains used to generate the tree are provided in Supplemental Materials: Table S1.

The genetic variability of USUV has been explored through phylogenetic studies performed over full-length sequences, as well as the envelope and NS5 genes [5,6,7,8,9]. These analyses cluster USUV sequences into distinct lineages which are designated on the basis of their geographic origin of isolation: Africa 1, 2 and 3 and Europe 1, 2, 3 and 4 (Figure 1). A recent phylogenetic study focused on partial NS5 gene sequences (265 bp) revealed a novel lineage (Europe 5) composed of strains isolated from birds in Germany in 2016 [8]. The prototype African strain SAAR-1776 (represented with a star in Figure 1) is distantly related to the European lineages. In addition to these genetic differences, SAAR-1776 differs from other USUV strains in that it was generated by intracerebral inoculation of newborn mice [1].

**Figure 2:**
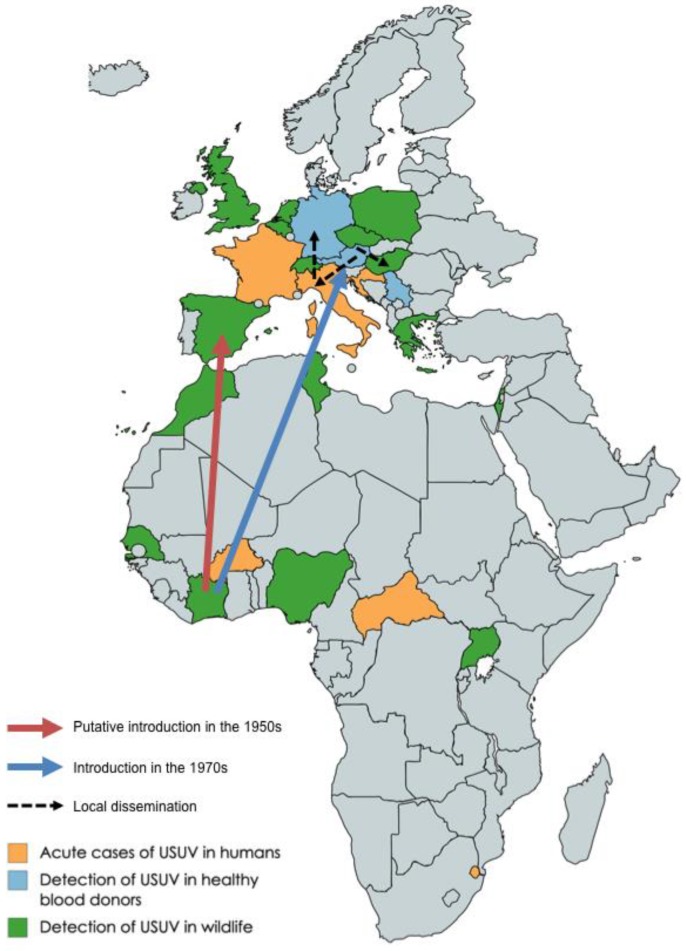
Geographical distribution of USUV. Countries where USUV was detected in wildlife (birds, mosquitoes, horses, etc.) are indicated in green; countries where USUV was detected in healthy blood donors are represented in blue; and countries where USUV caused symptomatic, acute infections are shown in orange. The arrows represent the presumed bird migration event that led to introduction of USUV in Europe, according to Engel et al. [7].

USUV is thought to have been introduced in Europe multiple times through bird migration, starting in the 1950s [7]. The Europe 1 lineage is thought to derive from a virus in Senegal that reached Spain, and is likely the origin of the first epizoosis in Central Europe. According to the same study, the Europe 2 and 3 lineages probably originated in Austria in 1993, and in Italy in 2007, respectively (Figure 2). Since these introductions, the virus has spread rapidly around the Mediterranean basin and Central Europe, reaching Tunisia [10], Morocco [11], Israel [12], Greece [13], France [14], Spain [15], Poland [16], Hungary [17], Czech Republic [18], Serbia [5], the United Kingdom [19], Croatia [20], the Netherlands [21], Switzerland [22], Italy [23], where it gave rise to the Europe 4 lineage, and Germany [24], where 3 different lineages have been recently shown to co-circulate [25]. Recent evidence suggests that introduction events into Europe from Africa are still happening. Indeed, viruses from African lineages were recently detected in Germany [26] and in the Camargue area of France [14].

The viral or immunological determinants responsible for USUV adaptation to birds and humans remain unclear. Similar to other arboviruses alternating between the insect vector and mammalian host species, evidence of strong purifying selection was detected across the entire viral genome [7]. In the case of USUV, European lineages seem to be subjected to a stronger negative selection than African lineages [7]. Nevertheless, one position in the viral polymerase gene NS5 (amino acid 898) evolved under significant positive selection, possibly reflecting an ongoing host/pathogen evolutionary conflict. Interestingly, another substitution in the NS5 gene has been observed in the Bologna/09 viral strain [27] derived from an USUV-positive patient recovering from orthotropic liver transplant [28]. As more viral sequences are made available, the viral determinants influencing USUV pathogenesis may be identified, as well as whether or not other factors such as evasion from the IFN response participate in this process.

## 3. Transmission Dynamics

### 3.1. WNV and USUV Co-Circulation and Co-Infection Dynamics

The transmission dynamics of arboviruses are generally influenced by biological and environmental factors, such as the identity and population density of vector and reservoir species, the extrinsic incubation period, humidity, temperature, host immunity, etc. In this regard, USUV shares a few common features with WNV: both viruses are mainly transmitted by *Culex* mosquitoes, with migratory birds acting as the major amplifying host (Table 1). Therefore, it is not surprising that in Europe, where WNV has also recently re-emerged, the two viruses co-circulate in 10 European countries and in 34 species of birds [29], as well as in horses [10]. Furthermore, 9 cases of birds [19,30] and 3 cases of humans [31,32] with positive serology for both viruses have been reported to date. This may be the result of local co-circulation of WNV and USUV, although cross-reactivity of antibodies complicates interpretation of results. Indeed, the envelope protein of USUV shares some structural features with the WNV envelope [33,34], and antibody cross-reactivity has been observed [35]. Thus, serological tests, such as ELISAs, cannot always distinguish USUV from WNV, which has led to the hypothesis that USUV circulation may be underestimated [36]. Other assays, such as protein microarray based on NS1 [37] or quantitative multiplex real-time RT-PCR [38], have been developed in recent years.

In one human case, viral RNA was detected simultaneously for both viruses [31,32], confirming that co-infection does occur. How significant USUV and WNV co-circulation and co-infections are at the population level, and whether a previous flavivirus infection influences subsequent WNV or USUV infections, remain to be determined. An *in vivo* study suggested that the immunization of mice with recombinant WNV particles induced low levels of antibodies cross-reacting with USUV [39]. Similarly, a previous infection with USUV was shown to reduce the susceptibility of adult mice to WNV [40]. Future work should determine whether such processes of cross-protection, or in the opposite sense, whether antibody enhancement of infection, may occur and be relevant for USUV transmission in birds and humans.

### 3.2. Vector Species for USUV

While USUV has been detected in a wide range of mosquitoes, it seems to be most frequently associated with *Culex pipiens* [41,42], which is thought to be the main vector for USUV in Europe. Indeed, competence studies have shown that *Culex pipiens* supports high levels of USUV infection [43,44,45,46]. Notably, competence of *Culex pipiens* for USUV and WNV is comparable at 18 °C and 23 °C, but not at 28 °C, where mosquitoes had higher susceptibility for USUV [43]. This may indicate some interesting temperature-related differences in the ability of the two viruses to overcome anatomical barriers and bottlenecks. As noted for other arboviruses, there may be some variation in the ability of USUV to infect different *Culex pipiens* populations. Indeed, the prototype strain SAAR-1776 could only infect one of the two *Culex pipiens* colonies from the United Kingdom [44], suggesting that host factors or genetics may restrict infection in these mosquitoes. Whether this is specific to the SAAR-1776 strain, or whether this happens with European USUV strains, remains to be established.

Other species of *Culex* have been described to be competent for USUV: for instance, both *Culex neavei* and *Culex quinquefasciatus* show high infection and dissemination rates [45,47]. USUV has been detected in pools of *Aedes albopictus* [41,48,49], an invasive mosquito species involved as an alternative vector for the transmission of many arboviruses, such as CHIKV. This prompted further investigation into *Aedes albopictus* competence for USUV. The results indicated that *Aedes albopictus* exhibits a lower competence for USUV [45], although the virus can replicate in the body and escape the midgut barrier [50]. In more rare cases, USUV was also detected in other mosquito species such as *Aedes japonicus, Aedes vexans*, *Anopheles maculipennis*, *Anopheles plumbeus, Coquillettidia richiardii*, *Culiseta annulata*, and some *Ochlerotatus* species [41,42,46]. USUV has never been detected in ticks, even in areas such as Italy where USUV circulation is known to be significant [51,52]. Since a single mutation was required for CHIKV adaptation to *Aedes albopictus* [53], sustained surveillance efforts may be required to rapidly detect potential adaptation of USUV to new vector species, which may allow viral dissemination into new geographic regions.

### 3.3. Reservoir of USUV

The main USUV natural hosts are birds, with infection being reported to date in 93 different species belonging to 35 families [54]. USUV is particularly pathogenic in a few species such as blackbirds (*Turdus merula*), gray owls (*Strix nebulosa*), and house sparrows (*Passer domesticus*) [17,55]. In these birds, systemic USUV replication is likely the cause of the severe pathogenesis, as the virus was detected in many organs such as the liver, heart, brain, and spleen [56]. Necrotic lesions were noted in these organs [57]. It is currently not known whether USUV can infect American robins (*Turdus migratorius*), a bird species that was critical for WNV dissemination across the US [58]. However, USUV potently infects its European counterpart *Turdus merula,* and many other *Passeriformes* birds known to be susceptible to WNV, such as the common starling (*Sturnus vulgaris*) or the house sparrow [29].

USUV induced mass mortality in blackbirds and grey owls in Austria [57], Germany [56], France [59], and the Netherlands [21], with dramatic consequences on bird populations. For instance, in Germany, the blackbird population declined by 15% within 5 years of USUV’s arrival [8]. In Austria, however, bird mortality sharply dropped after 2004, while the number of infected birds with low viral titers increased [17]. This may have resulted from the acquisition of herd immunity: in owls and birds of prey, the proportion of seropositive birds reached levels above 50% [60]. Interestingly, only a relatively low number of bird species have been subjected to large die-off episodes, and in some avian species such as chickens or geese, experimental inoculation of USUV only caused mild pathogenicity [61,62]. The basis for these species-specific differences in pathogenicity is currently unknown. The co-infection of birds with USUV and other pathogens may increase disease severity: for instance, co-infection of birds with USUV and *Plasmodium* spp. can lead to a more severe outcome than when they carry only one of these pathogens [21,63]. Future investigation will help determine which virological and immunological factors influence USUV pathogenesis and co-morbidities.

### 3.4. Other Dead-End Hosts

Beyond infection of birds and humans, USUV has been detected in many mammalian species considered to be dead-end hosts. As is the case with WNV, horses are considered to be a sensitive host for USUV: viral circulation has been detected in Tunisia [10], Spain [64], Serbia [65], Poland [16] and Croatia [20]. USUV can also infect dogs [66,67], dear [68] and wild boars [69]. USUV was also detected in bats [70], squirrels [71] and rodents [72], in which the virus did not seem to cause the same severe pathogenicity as it does in birds, raising concerns that these species could act as secondary reservoirs. Whether USUV can reach a viremia in these animals to sustain a new mosquito infection cycle is unclear. The expanding range of wild animals susceptible to USUV highlights its potential as an important emerging pathogen.

## 4. Human Clinical Cases and Disease

The first human cases of USUV infection were described in Central African Republic and Burkina Faso in the 1980s and in 2004, respectively, with mild symptoms such as fever and rash [2]. To date, a total of 28 acute USUV infections have been reported in humans [73], including some serious complications such as meningoencephalitis [28,74] and facial paralysis [75]. Seroprevalence studies suggested that USUV infections in humans may have been largely underestimated, and many of them may be asymptomatic. Indeed, antibodies against USUV were detected in the blood of 0.01% to 1% of healthy blood donors in Germany and Italy, respectively [49,76,77]. Higher seroprevalence was detected in serum samples collected from healthy individuals in Serbia (7.5%) [78] and from forestry workers in Italy (18%) [79]. A large retrospective study, conducted on over 900 patients, of which about a third were suspected for encephalitis or meningoencephalitis, found a prevalence of 6.5% [80]. In contrast, two studies failed to detect USUV in the cerebrospinal fluid of patients with encephalitis in Italy and Switzerland [81,82]. These studies indicate that USUV already circulates to significant levels in human populations, although it is rarely associated with neurological complications.

While most USUV infections are mild or asymptomatic, neurotropism represents a growing concern for human health, especially since two immuno-compromised patients tested positive for USUV. One of them was recovering from a liver transplant [28], and the other was diagnosed with a B cell lymphoma [74]: both showed signs of neuropathogenesis that were thought to be caused by USUV. Both survived the infection. USUV RNA was also detected by RT-PCR in cerebrospinal fluid of patients suffering from meningoencephalitis, suggesting that the virus may establish an infection in the brain [83]. Several other lines of evidence suggest that USUV can be neurotropic. First, this virus was detected in the brains of dead bats and birds in the wild [57,70]. Furthermore, experimental infection of 1-week-old suckling mice leads to paraplegia and paralysis, and is associated with apoptosis and demyelination of neuronal and glial cells [84]. Moreover, the USUV virus isolated from a patient diagnosed with facial paralysis could infect primary astrocytes [75]. An *in vitro* study confirmed these findings and demonstrated that USUV can establish productive infection and induce apoptosis in a wide range of neural cells such as neurons, astrocytes, microglial cells and neuronal stem cells [85]. USUV was even shown to be associated with levels of infection and apoptosis in neuronal cells that were higher than ZIKV, suggesting that USUV may potentially cause significant neurological defects [85].

## 5. Cellular Responses to USUV Infection

USUV infection triggers the induction of pro-inflammatory and antiviral responses, including cytokine secretion. In primary human nasal epithelial cells, USUV induces a modest expression of IL6, IL8 and IP10, albeit to lower levels than JEV [86]. In dendritic cells (DCs), USUV induces high levels of TNF-α [87], and of both IFN-α and IFN-β [87,88], leading to the expression of Interferon Stimulated Genes (ISGs). Interestingly, cells infected by USUV induce IFN to higher levels than cells infected by WNV (10- to 100-fold, depending on the multiplicity of infection and time point considered) [88]. USUV is also very sensitive to the antiviral effect of IFN: in A549 cells, USUV replication is restricted 10 times more potently than WNV by a wide variety of IFN-I and IFN-III subtypes [88]. Consistent with these findings, while adult Swiss mice are highly susceptible to WNV infection, they are not to USUV [40]. In contrast, USUV infection induced high mortality rates in suckling mice, which have not yet developed a functional IFN response [40,84], or in AG129 mice, knocked-out for the IFN-α and IFN-γ pathways [89]. Taken together, these results suggest that USUV is potently controlled by the IFN response, which may account (at least in part) for its low viral pathogenicity in humans.

Conversely, USUV may also hijack cellular responses to its advantage: for instance, it triggers the autophagy pathway in infected cells, stimulating viral replication [90]. The interplay between autophagy and flaviviruses, which has been described before [91], could be particularly interesting, since it may provide cellular drug targets. Indeed, the autophagy inhibitors 3-methyladenine and wortmanin significantly reduced USUV replication in Vero cells (from 3-fold to 5-fold) [90]. Host lipid biosynthesis pathways are also required for the production of infectious viral particles: inhibition of the acetyl-CoA carboxylase (ACC) enzyme by two different drugs very potently (up to 3 logs) inhibited WNV and USUV [92]. Finally, favipiravir, a broad-spectrum viral RNA polymerase inhibitor, has shown some efficacy in restricting USUV replication in the AG129 model [89].

## 6. USUV Circulation in the Future: Surveillance and Mathematical Modeling

The circulation of USUV in bird and human populations is already solidly established in Europe, as evidenced by the massive decline in blackbird populations [8] and the significant levels of seroconversion in human blood donors [76,77,78,79,80]. However, the extent of USUV circulation in 2018 is unprecedented [93]. In France, USUV was detected from dead birds in 46 administrative districts, versus only 4 in 2017 (Cécile Beck, personal communication). In Austria, 18 blood donors (16 of them asymptomatic) tested positive for USUV, while only 6 did in 2017 [32]. According to the European Centre for Disease Prevention and Control (EDCC), WNV circulation also reached historically high levels in Europe in 2018, with 2083 human cases, representing a 7-fold increase compared to 2017. Some countries, such as Bulgaria (15-fold increase), France (13.5-fold increase) and Italy (11-fold increase) were particularly impacted by the uptick in WNV circulation. A likely hypothesis for this is that some environmental and ecological factors influencing both USUV and WNV account for the exceptional levels of viral circulation in 2018. However, the possibility that viral adaptation may have contributed to this increased circulation of USUV cannot be ruled out. Therefore, surveillance of USUV circulation, as well as monitoring USUV evolution, will be crucial to try to prevent future outbreaks. This goal remains difficult to achieve, notably because of the lack of large-scale cohort studies and the absence of commercially available diagnostic reagents for USUV. In this context, mathematical modeling can help estimate the potential levels of USUV circulation. Habitat models have recently been used to predict the number of USUV cases in under-sampled areas [94]. Two models have been developed to study USUV transmission dynamics: a mechanistic epidemiological model, aimed at estimating USUV transmission in Austria [95,96]; and a correlation-based environmental model, based on data collected in Germany [8]. Both models were used in a recent study to generate risk maps for USUV [97]. Large areas of Central and Western Europe including northern Italy, Austria, southern France, and a large area spanning the North East of France, Belgium, and the North West of Germany, were predicted as suitable for USUV replication. As temperatures are likely to increase in Europe because of the effects of climate change, these risk maps are even likely underestimating future USUV transmission rates.

Given the similarities in the ecology of WNV and USUV (Table 1), and the relatively recent emergence of USUV in Europe, it is possible that USUV has the potential to reach levels of viral circulation similar to those of WNV. For now, USUV has seemingly not reached the United States, but a study established that North American colonies of *Culex pipiens* and *Culex quinquefasciatus* are competent vectors for USUV [45]. As stated earlier, USUV also infects multiple bird species very closely related to the ones that were involved in dissemination of WNV in the United States. Therefore, USUV emergence outside of Europe should be considered a real possibility, prompting research and surveillance efforts.

On the other hand, it is possible that WNV and USUV are competing with each other, and that the co-existence of both viruses at high levels in the same geographical areas and ecological niches is impossible. Such competition between two related flaviviruses has been observed before, for instance in the case of WNV and Saint Louis encephalitis virus (SLEV). Indeed, prior to WNV emergence in the US, SLEV was endemic in southern California, but circulation drastically dropped after WNV invasion in 2003 [104]. This led to the hypothesis that SLEV, while still capable of sporadically re-emerging [105], has been outcompeted by WNV, which infects similar bird and vector species with greater success. 

## 7. Concluding Remarks

While USUV has caused significant outbreaks in birds since 2001, its limited circulation and overall low pathogenicity in humans explain why researchers and public health authorities are not yet too concerned about this pathogen. In the context of unprecedented viral circulation in 2018, and since USUV has now clearly been shown to be associated with neurological disorders, increased surveillance and research efforts are in order. In particular, larger cohort studies of populations of interest, such as wildlife workers and patients suffering from encephalitis, will help more accurately estimate USUV prevalence. Identifying molecular determinants associated with virulence, host tropism, and adaptation to new vectors may help anticipate key events leading to the possible emergence of USUV. Finally, developing antiviral approaches, or other preemptive or control measures in humans or at the reservoir-vector interface, will help control USUV circulation and reduce the economic and sanitary burden it may pose in the future.

## Figures and Tables

**Table 1:** Comparison of WNV and USUV main characteristics. Some important features of West Nile Virus (WNV) and Usutu Virus (USUV) are listed here, with the corresponding bibliographical references.

	WNV	USUV
**Geographical Distribution**	Africa, Europe, Middle East, North America and West Asia	Africa, Europe
**Main Vector**	*Culex* spp.	*Culex* spp.
**Putative Seconday Vector**	*A. vexans* [98], *A. japonicus* [99], *A. albopictus* [100]	*A. albopictus* [45,50]
**Amplifying Host**	Migratory birds	Migratory birds
**Main Route of Transmission**	Mosquito bite	Mosquito bite
**Alternate Route of Transmission**	Rare cases of contamination through organ transplant and transfusion [101] and of mother-to-child transmission [102]	Not described
**Sensitivity to IFN**	Strong [88]	Very strong [88]
**Antagonism of IFN Response**	Through NS4B [103]	Not described

## Supplementary Materials

The following are available online at https://www.mdpi.com/1999-4915/11/7/640/s1, Table S1: List of USUV strains used to build the phylogenetic tree.
